# Gender Disparities in the Presentation, Management and Outcomes of Acute Coronary Syndrome Patients: Data from the 2nd Gulf Registry of Acute Coronary Events (Gulf RACE-2)

**DOI:** 10.1371/journal.pone.0055508

**Published:** 2013-02-06

**Authors:** Abdulla Shehab, Bayan Al-Dabbagh, Khalid F. AlHabib, Alawi A. Alsheikh-Ali, Wael Almahmeed, Kadhim Sulaiman, Ahmed Al-Motarreb, Nicolaas Nagelkerke, Jassim Al Suwaidi, Ahmad Hersi, Hussam Al Faleh, Nidal Asaad, Shukri Al Saif, Haitham Amin

**Affiliations:** 1 Department of Internal Medicine, Faculty of Medicine and Health Sciences, United Arab Emirates University, Al Ain, United Arab Emirates; 2 King Fahad Cardiac Center, College of Medicine, King Saud University, Riyadh, Kingdom of Saudi Arabia; 3 Heart and Vascular Institute, Sheikh Khalifa Medical City, Abu Dhabi, United Arab Emirates; 4 Institute for Clinical Research and Health Policy Studies and Tufts University School of Medicine, Boston, Massachusetts, United States of America; 5 Department of Cardiology, Royal Hospital, Muscat, Oman; 6 Faculty of Medicine, Sana’s University, Sana’a, Yemen; 7 Department of Community Medicine, Faculty of Medicine and Health Sciences, United Arab Emirates University, Al Ain, United Arab Emirates; 8 Department of Cardiology and Cardiovascular Surgery, Hamad Medical Corporation (HMC), Doha, Qatar; 9 Department of Cardiac Sciences, College of Medicine, King Saud University, Riyadh, Saudi Arabia; 10 Cardiology Department, Saud Al-Babtain Cardiac Center, Dammam, Saudi Arabia; 11 Cardiology Department, Mohammed Bin Khalifa Cardiac Center, Manama, Bahrain; Universidad Peruana de Ciencias Aplicadas (UPC), Peru

## Abstract

**Background:**

Gender-related differences in mortality of acute coronary syndrome (ACS) have been reported. The extent and causes of these differences in the Middle-East are poorly understood. We studied to what extent difference in outcome, specifically 1-year mortality are attributable to demographic, baseline clinical differences at presentation, and management differences between female and male patients.

**Methodology/Principal Findings:**

Baseline characteristics, treatment patterns, and 1-year mortality of 7390 ACS patients in 65 hospitals in 6 Arabian Gulf countries were evaluated during 2008–2009, as part of the 2nd Gulf Registry of Acute Coronary Events (Gulf RACE-2). Women were older (61.3±11.8 vs. 55.6±12.4; P<0.001), more overweight (BMI: 28.1±6.6 vs. 26.7±5.1; P<0.001), and more likely to have a history of hypertension, hyperlipidemia or diabetes. Fewer women than men received angiotensin-converting enzyme inhibitors (ACE), aspirin, clopidogrel, beta blockers or statins at discharge. They also underwent fewer invasive procedures including angiography (27.0% vs. 34.0%; P<0.001), percutaneous coronary intervention (PCI) (10.5% vs. 15.6%; P<0.001) and reperfusion therapy (6.9% vs. 20.2%; P<0.001) than men. Women were at higher unadjusted risk for in-hospital death (6.8% vs. 4.0%, P<0.001) and heart failure (HF) (18% vs. 11.8%, P<0.001). Both 1-month and 1-year mortality rates were higher in women than men (11% vs. 7.4% and 17.3% vs. 11.4%, respectively, P<0.001). Both baseline and management differences contributed to a worse outcome in women. Together these variables explained almost all mortality disparities.

**Conclusions/Significance:**

Differences between genders in mortality appeared to be largely explained by differences in prognostic variables and management patterns. However, the origin of the latter differences need further study.

## Introduction

Coronary artery disease (CAD) is a leading cause of death and disability worldwide [1]. Its acute clinical manifestation in the form of Acute Coronary Syndrome (ACS) can present as unstable angina (UA), non-ST-elevation myocardial infarction (NSTEMI) or ST-elevation myocardial infarction (STEMI) [2], [3]. Gender differences have been reported in the presentation, management, and prognosis of patients with ACS [4]–[7] with women typically having a more adverse prognosis than men. Yet, the role of gender in ACS remains controversial, specifically as to whether differences in outcomes can be explained by differences in baseline risk factor characteristics at presentation or the acute management of ACS. For example, several studies have shown that fewer women with ACS undergo coronary angiography or timely revascularization [8]–[14]. In contrast, other studies have shown negligible gender bias in the management of ACS [15], [16]. Significant gender differences in both management and outcomes of ACS patients have been reported from the first Gulf Registry of Acute Coronary Events (Gulf RACE) [17]. To better understand these differences, specifically in 1-year mortality, we explored to what extent gender outcome disparities can be attributed to demographic, baseline clinical risk factors, and management differences between female and male patients. In the present analysis, we thus describe gender differences in presentation, management and outcomes of patients with ACS using the most recently completed multinational ACS registry in the Middle East, and explore whether such potential disparities can be explained by gender differences in risk factors or acute management of ACS.

## Methods

### Patients and Data Collection

We used data from Gulf RACE-2, a prospective multinational, multicentre registry of patients above 18 years of age hospitalized with the final diagnosis of ACS, including UA, NSTEMI and STEMI from 65 hospitals in 6 Middle Eastern countries (Bahrain, Saudi Arabia, Qatar, Oman, United Arab Emirates (UAE), and Yemen). Details of Gulf RACE-2 have been previously described [18]. There were no patient-specific exclusion criteria, and patient recruitment occurred from October 2008 until June 2009. On-site cardiac catheterization laboratories and coronary care units were available in 43% and 71% of hospitals, respectively. A case report form for each patient with suspected ACS was filled out upon hospital admission by assigned physicians and/or research assistants working in each hospital using standard definitions, and standardized follow-up information was collected throughout the patient’s hospital stay. All case report forms were verified by a cardiologist and then submitted online (www.gulf-acs.com) to the principal coordinating center, where they underwent further checks before submission for final analysis.

The protocols of the study were approved by the institutional ethical review boards of all the countries participating hospitals. The Gulf RACE-2 lists all 65 hospitals that provided data for this multicenter study. Informed verbal consent was taken from the patients before enrolling them into the study. Written consent was not required by the ethics committees since it was an observational study. Measures were taken to ensure this process through communication with the cardiologist supervising each hospital. The data is not from a publicly accessible site. Definitions of the sets of variables collected from the patients, outcome parameters as well as the diagnosis of ACS types followed the American College of Cardiology clinical data standards [19].

### Statistical Analysis

Data of all 7930 patients were analyzed with SPSS statistical software version 19.0 (Chicago, Illinois, USA) and genders were compared for clinical characteristics, management, in-hospital outcomes and 30-day and 1-year post discharge mortality. Continuous variables were expressed as mean ± SD and were compared between men and women using the Student t-test. Categorical variables were expressed by their frequency distributions and were compared using the Pearson’s chi-square tests or Fisher’s exact test. Multivariable logistic regression, stepwise with backward variable selection, was performed to evaluate the association between gender and 1-year mortality while adjusting for confounding and, where appropriate, management related variables. After variable selection, models were rerun with gender (whether selected or not) and the selected other variables. Potential confounders were prognostic variables that differed at baseline, between male and female patients, such as age and a history of diabetes mellitus (DM). Confounding variables were divided into 2 levels, *viz.* i) demographic: age, country; ii) demographic and comorbidities: age, country, diagnosis, Killip class, predominant presenting symptoms, history of: congestive heart failure (CHF), hypertension, hyperlipidaemia, and DM, smoking and body mass index (BMI). Stepwise logistic regression (Model II and III) was used to adjust for each of these levels of confounding variables. Management variables, which were added in regression model IV, were invasive procedures: reperfusion therapy, percutaneous coronary intervention (PCI), and coronary artery bypass graft (CABG) and medications at discharge (aspirin, statins, beta blockers (BB), calcium channel blockers (CCB), angiotensin-converting enzyme (ACE) inhibitors, angiotensin II receptor blockers (AIIRB) and clopidogrel) that could have been influenced by the gender of the patient. All P-values <0.05 (2-sided) were considered statistically significant. As, due to loss to 1-year follow-up, crude1-year mortality rates may be somewhat biased (ascertainment of deads is higher than of 1-year survivors), we also calculated 1-year survival/mortality rates by multiplying the probability of surviving hospital, times the probability of surviving until 1 month discharge times probability to surviving 1 year if alive 1 month after discharge.

## Results

### Baseline Characteristics

Females comprised 21.3% (1686/7930) of ACS patients in the database. The clinical characteristics of the study population are shown, broken down by gender, in Table 1.

**Table 1:** Baseline characteristics of patients stratified by gender (n = 7930).

Variable	Men(n = 6244)	Women(n = 1686)	P-value
**Age**, mean±SD, years	55.6±12.4	61.3±11.8	<0.001
**BMI**, mean±SD, kg/m^2^	26.7±5.1	28.1±6.6	<0.001
**Medical history**			
Hypertension	2639 (42.3)	1107 (65.7)	<0.001
Hyperlipidemia	1886 (30.2)	711 (42.2)	<0.001
Diabetes mellitus	2243 (35.9)	892 (52.9)	<0.001
Smoking	2722 (43.6)	108 (6.4)	<0.001
Family history of CAD	659 (10.6)	151 (9.0)	0.06
Angina	2217 (35.5)	805 (47.7)	<0.001
MI	1190 (19.1)	336 (19.9)	0.423
PCI	570 (9.1)	157 (9.3)	0.812
CABG	257 (4.1)	79 (4.7)	0.306
CHF	353 (5.7)	170 (10.1)	<0.001
**Predominant presenting symptoms**			<0.001
Ischemic type chest pain	5387 (86.3)	1283 (76.1)	
Atypical chest pain	292 (4.7)	107 (6.3)	
Dyspnea	350 (5.6)	208 (12.3)	
Fatigue	11 (0.2)	6 (0.4)	
Loss of consciousness	44 (0.7)	13 (0.8)	
Cardiac arrest/aborted sudden death	35 (0.6)	12 (0.7)	
Palpitation	37 (0.6)	16 (0.9)	
Other	88 (1.4)	41 (2.4)	
**ACS diagnosis**			<0.001
STEMI/MI	3110 (49.8)	503 (29.8)	
NSTEMI/UA	3134 (50.2)	1183 (70.2)	
**Killip class** *			<0.001
I	4936 (79.1)	1173 (69.9)	
II	842 (13.5)	311 (18.4)	
III	278 (4.5)	130 (7.7)	
IV	187 (3.0)	72 (4.3)	
**Country**			<0.001
Saudi Arabia	1780 (28.5)	386 (22.9)	
Bahrain	458 (7.3)	127 (7.5)	
Yemen	1394 (22.3)	367 (21.8)	
Oman	1458 (23.4)	654 (38.8)	
UAE	508 (8.1)	89 (5.3)	
Qatar	646 (10.3)	63 (3.7)	

Figures in parentheses are percentages and continuous variables are shown as mean±SD.

Abbreviations: SD, standard deviation; BMI, body mass index; CAD, coronary artery disease; MI, myocardial infarction; PCI, percutaneous coronary intervention; CABG, coronary artery bypass graft; CHF, congestive heart failure; STEMI, ST elevation myocardial infarction; NSTEMI, non-ST-elevation myocardial infarction; UA, unstable angina.

* Killip class (scale I–IV) a system used to stratify the severity of left ventricular dysfunction and determines clinical status of patients post myocardial infarction (MI).

Killip classification:

Class 1: No rales, no 3rd heart sound.

Class 2: Rales in <1/2 lung field or presence of a 3rd heart sound.

Class 3: Rales in >1/2 lung field–pulmonary edema.

Class 4: Cardiogenic shock–determined clinically.

Females were older, had higher BMI, and were diagnosed more frequently with NSTEMI/UA (70.2% vs. 50.2%, P<0.001). Female patients also had higher prevalence of hypertension, diabetes and hyperlipidemia (P<0.001 for all comparisons). Atypical chest pain was more common in women (6.3% vs. 4.7%, P<0.01). Smoking was less prevalent among women (6.4% vs.43.6%, P<0.001) than men, and women were more likely to present with Killip class II, III or IV (30.1% vs. 20.9%, P<0.001).

### Medical Treatments Received

Table 2 shows the frequency of use (within 24 hours of admission) of the different drugs by gender. Both groups equally received aspirin, but, males had a higher prescription rate of clopidogrel (79.2% vs. 64.9%; P<0.001) and BBs (75.4% vs. 70.6%; P<0.001), while females received more CCB (11.0% vs. 6.2%; P<0.001), AIIRBs (7.8% vs. 4.2%; P<0.001), insulin (38.0% vs. 27.3%; P<0.001) and oral hypoglycemic agents (OHA) (10.6% vs. 6.5%; P<0.001).

**Table 2:** Treatment on admission and at discharge of the study cohort stratified according to gender (n = 7930).

	Men(n = 6244)	Women(n = 1686)	P-value
**Admission medical treatment**			
Aspirin	6145 (98.4)	1656 (98.2)	0.588
Clopidogrel	4947 (79.2)	1094 (64.9)	<0.001
BB	4705 (75.4)	1190(70.6)	<0.001
CCB	390 (6.2)	186 (11.0)	<0.001
ACE	4420 (70.8)	1184 (70.2)	0.651
AIIRB	262 (4.2)	132 (7.8)	<0.001
Statins	5936 (95.1)	1578 (93.6)	0.019
Insulin	1707 (27.3)	641 (38.0)	<0.001
OHA	406 (6.5)	179 (10.6)	<0.001
**In-hospital invasive procedures**			
Cardiac angiography	2120 (34.0)	456 (27.0)	<0.001
PCI	971 (15.6)	177 (10.5)	<0.001
CABG	193 (3.1)	40 (2.4)	0.125
Reperfusion therapy	1259 (20.2)	117 (6.9)	<0.001
**Discharge medical treatment**			
Aspirin	5834 (93.5)	1523 (90.4)	<0.001
Clopidogrel	4439(71.1)	924 (54.8)	<0.001
BB	5020 (80.6)	1248 (74.3)	<0.001
CCB	415 (6.7)	203 (12.1)	<0.001
ACE	4501 (72.3)	1140 (67.9)	<0.001
AIIRB	369 (5.9)	167 (9.9)	<0.001
Statins	5735 (92.1)	1482 (88.2)	<0.001
Insulin	747 (12.0)	374 (22.3)	<0.001
OHA	1486 (23.9)	513 (30.5)	<0.001

Figures in parentheses are percentages. Abbreviations: BB, beta-blockers; CCB, calcium channel blockers; ACE, angiotensin-converting enzyme inhibitors; AIIRB, angiotensin II receptor blockers; OHA, oral hypoglycemic agents; PCI, percutaneous coronary intervention; CABG, coronary artery bypass graft.

More men than women underwent in-hospital revascularization procedures, specifically PCI (15.6% vs. 10.5%; P<0.001) and reperfusion therapy (20.2% vs. 6.9%; P<0.001). At discharge, men were more likely prescribed aspirin (93.5% vs. 90.4%; P<0.001), clopidogrel (71.1% vs. 54.8%; P<0.001), BBs (80.6% vs. 74.3%; P<0.001), ACE (72.3% vs. 67.9%; P<0.001) and statins (92.1% vs. 88.2%; P<0.001), while women were more likely to be treated with CCBs (12.1% vs. 6.7%; P<0.001), AIIRBs (9.9% vs. 5.9%; P<0.001), insulin (22.3% vs. 12.0%; P<0.001) and OHAs (30.5% vs. 23.9%; P<0.001).

### Clinical Outcomes and Mortality

**Figure 1:**
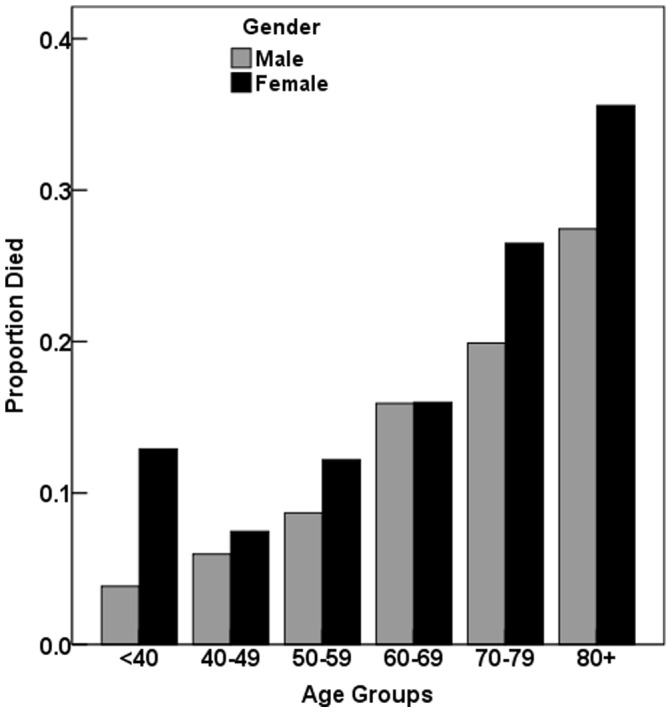
Proportion of patients dying in-hospital and within one year from hospital discharge (n = 6132). Risk of mortality significantly increased with age (P<0.001; by logistic regression); the interaction between gender and age was not statistically significant (P = 0.70; by logistic regression).

**Figure 2:**
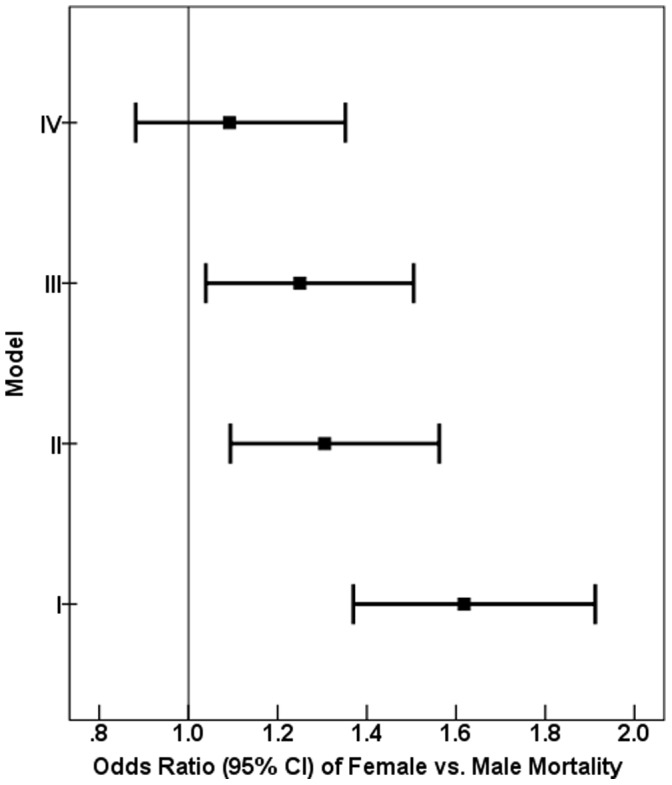
Association of gender (female) and mortality derived from multivariate-adjusted analyses (n = 7930). Model I included gender only (n = 6132), (OR = 1.62; 95% CI: 1.37–1.91; P<0.001). Model II was adjusted for gender, age and country (n = 6132), (OR = 1.31; 95% CI: 1.09–1.56; P = 0.003). Model III was adjusted for gender, age, country, diagnosis, Killip class, predominant presenting symptoms, history of CHF and DM (n = 5956), (OR = 1.25; 95% CI: 1.04–1.51; P = 0.018). Smoking, BMI, history of hypertension and hyperlipidaemia were considered but removed because of non-significant associations. Model IV was adjusted for gender, age, country, diagnosis, Killip class, predominant presenting symptoms, history of CHF and DM, discharge medication, including: aspirin, statins, BBs, CCBs, ACE, AIIRBs, and reperfusion (n = 5934), (OR = 1.09; 95% CI: 0.88–1.35; P = 0.417). Smoking, BMI, history of hypertension and hyperlipidaemia, clopidogrel as discharge medication, PCI, and CABG were considered but removed because of non-significant associations. *Predominant presenting symptoms includes: ischemic type chest pain, atypical chest pain, dyspnea, fatigue, loss of consciousness, cardiac arrest/aborted sudden death, palpitation and other symptoms. CI = confidence interval.

There were several differences in non-fatal in-hospital outcomes such as recurrent ischemia (14.6% vs. 19.2%), CHF (11.8% vs. 18%), ventilation (4.1% vs. 6.7%), cardiogenic shock (5.6% vs. 8%) (P<0.001 in all cases), but not in re-infarction (2.0% vs. 2.6%), stroke (0.7% vs. 0.8%) and major bleeding (0.5% vs. 0.8%) between men and women. While in-hospital death (yes/no) was recorded for all 7930 patients, whether patients were dead or alive at year 1, was only ascertained for 6132 patients. Mortality as estimated by crude mortality was only slightly higher than that estimated using the multiplicative method (Table 3). Ascertainment of 1-year mortality also differed by country, ranging from 69.3% in Oman to 92.1% in the UAE. The unadjusted incidences of in-hospital death (6.8% vs. 4%, P<0.001), 1-month post discharge death (11.0% vs. 7.4%, P<0.001) and 1-year post discharge death (17.3% vs. 11.4%, P<0.001) were higher in women than in men (Table 3). There were considerable, statistically significant, differences in crude 1-year mortality rates among countries with the highest crude mortality observed in Yemen. Gender differences were also not homogeneous among countries (Breslow-Day’s test for homogeneity of odds ratios P = 0.03). Figure 1 shows the 1-year total mortality risk by gender broken down by age group, and shows that gender disparities in mortalities exist at all ages. Figure 2 shows the odds ratio (OR) for the association between gender and 1-year mortality after adjustment for various levels of potential confounders, and shows the extent to which gender disparities are accounted for by the different levels of covariables (demographic, baseline risk factors, management). The unadjusted OR of female vs. male mortality was 1.62 (95% CI: 1.37–1.91; P<0.001). To explain this unadjusted difference (model I), a total of three multiple logistic regression were carried out: the first (model II) included demographics, i.e. gender, age and country only (n = 6132), (OR = 1.31; 95% CI: 1.09–1.56; P = 0.003); the second regression (model III) adjusted the effects of gender for age, country, diagnosis, BMI, Killip class and predominant presenting symptoms (n = 5956), (OR = 1.25; 95% CI: 1.04–1.51; P = 0.018); the third regression (model IV) adjusted the effects of gender for age, country, diagnosis, BMI, Killip class, tobacco smoking, predominant presenting symptoms, medical history, invasive procedures (reperfusion, PCI, CABG) and medications at discharge (n = 5934), (OR = 1.09; 95% CI: 0.88–1.35; P = 0.417). Results of the regression of model 4 are shown in Table 4.

**Table 3:** In-hospital outcomes and 1-month and 1-year post discharge mortality of the study cohort by gender (n = 7930).

Variable	Men(n = 6244)	Women(n = 1686)	P-value
Recurrent ischemia	910 (14.6)	323 (19.2)	<0.001
Infarction/Re-infarction	128 (2.0)	44 (2.6)	0.098
Congestive heart failure	738 (11.8)	303 (18.0)	<0.001
Ventilation	257 (4.1)	113 (6.7)	<0.001
Cardiogenic shock	347 (5.6)	135 (8.0)	<0.001
Stroke	42 (0.7)	14 (0.8)	0.293
Major bleed	34 (0.5)	14 (0.8)	0.124
Death, in-hospital	247 (4.0)	115 (6.8)	<0.001
Death, 1-month	406/5493 (7.4)	167/1525 (11.0)	<0.001
Death, 1-yearEstimated 1 year mortality	546/4782 (11.4)9.86%	233/1350 (17.3)15.27%	<0.001<0.001

Figures in parentheses are percentages.

**Table 4:** Variables in model IV, and their effects on mortality.

Variable	OR	95% CI	P-value
Female (vs. male)	1.09	0.88–1.35	0.42
Age	1.04	1.03–1.05	<0.001
Country			<0.001
Diagnosis			<0.001
Killip class I vs. II–IV	0.58	0.47–0.73	<0.001
History of CHF	1.48	1.07–2.03	0.018
History of DM	1.48	1.21–1.81	<0.001
Predominant symptoms*			0.001
**Discharge medications**			
Aspirin	0.26	0.19–0.36	<0.001
Statins	0.34	0.25–0.46	<0.001
BB	0.57	0.46–0.72	<0.001
CCB	0.59	0.40–0.86	0.006
ACE	0.64	0.51–0.81	<0.001
ARIIB	1.62	1.10–2.39	0.015
Reperfusion	0.66	0.48–0.91	0.011
Nagelkerke R^2^			0.373

ORs for variables with multiple levels (country, diagnosis, and predominant symptoms) are not shown. The variables that were dropped out of the multivariable logistic regression using the stepwise-backward elimination method included: smoking, BMI, history of hypertension and hyperlipidaemia, clopidogrel as discharge medication, PCI, and CABG.

* Predominant presenting symptoms includes: ischemic type chest pain, atypical chest pain, dyspnea, fatigue, loss of consciousness, cardiac arrest/aborted sudden death, palpitation and other symptoms.

Abbreviations: OR, odds ratio; CI, confidence interval; BB, beta-blockers; CCB, calcium channel blockers; ACE, angiotensin-converting enzyme inhibitors; AIIRB, angiotensin II receptor blockers.

## Discussion

The underlying pathology of ACS differed between the genders, with more women than men (70.2% vs. 50.2%, P<0.001) having NSTEMI/UA, while STEMI/MI was predominant in men (49.8% vs. 29.8%, P<0.001). Yet, women had notably higher unadjusted mortality rates, both in-hospital and post discharge, than men. Mortality risk strongly increases with age (Figure 1) and gender differences appear to exist at all ages.

Cardiovascular risk factors also differed between men and women. Our study is consistent with several studies showing that among patients with ACS, hypertension, DM and hyperlipidemia are more prevalent in women than in men [20], whereas men smoke more [21].

Women were generally treated more conservatively: women with ACS were less likely to undergo PCI and reperfusion than men (P<0.001). There are several factors that could explain the (relative) under-treatment of female patients with ACS with invasive cardiac procedures, such as being older, co-morbidities (hypertension, hyperlipidemia, diabetes), atypical ACS presenting symptoms [4], patients’ preferences not to undergo invasive cardiac procedures [13], [22]–[24], and physician’s fear of treatment outcome, especially regarding cardiac catheterization [13].

In addition to differences in invasive procedures, medications prescribed at discharge also differed. Men were prescribed significantly more statins, ACE inhibitors, clopidogrel, and beta-blockers but the absolute differences were modest. These results are consistent with other studies [25], [26]. In contrast, more women were taking insulin and OHAs at admission and discharge, but this may well reflects their higher prevalence of diabetes. In this context it may be interesting to note that various discharge medications appeared to be associated with either higher or lower mortality. However, as indications for choice of discharge medications were not recorded, it is unclear whether these associations have any bearing on the pharmaceutical effects of these medications in these patients or whether this reflects confounding by indication.

Our study is in accordance with previous studies where investigators have reported that women with NSTEMI/UA, compared to men, are in general older, have more co-morbidities at presentation, and undergo fewer invasive procedures [27]–[29]. As these differences together appear to account for most of the gender disparities in mortality, studies that look into the reasons for these differences are badly needed. For example, the age difference between male and female patients could be due to demographics, the protective effects of pre-menopause, or reluctance in seeking care. Our results also agree with our recent study on gender-related differences in ACS patients in the UAE only [30]. In the latter study, we analyzed a subset (n = 1697) of the first Gulf RACE data collected in 2007 of ACS patients at 18 UAE hospitals. We found that women were significantly older; more often had cardiac risk factors and were significantly less frequently treated with beta-blockers and reperfusion therapy. HF and adjusted mortality rates of women were higher in females compared with men [30].

Our 3 multivariate regression models (IIIV) indicate that most of the differences in mortality can be explained by both differences in potentially confounding baseline variables and, to a smaller extent, differences in medical management. Adjustment for baseline variables reduced an OR from 1.62 (model I) to 1.25 (model III) while adding management variables further reduced gender disparity to an OR of 1.09 (model IV). Of course, discrepancies in management are potentially amenable to interventions, while this is doubtful with regards to baseline variables. Thus, changing and standardizing guidelines can be expected to lead to some reduction in gender disparity in mortality outcome but not lead to its complete elimination.

The strengths of this study include its multi-national perspective, the complete spectrum of ACSs experienced by the large number of patients enrolled and the use of standardized criteria for defining ACS and hospital outcomes. However, the basic limitations of an observational registry-type study still apply, such as unidentified confounders which could influence the results, as well as some missing information on after discharge outcome, specifically mortality, which could potentially introduce some bias in our findings; specifically as it gives patients who succumbed (early) a higher probability of having complete information than those who survived (longer).

### Conclusion

Women patients with ACS are still more likely to be treated conservatively than their male counterparts. Apart from smoking, CVD risk factors, including obesity, hypertension, hyperlipidemia and diabetes are more prevalent in Middle Eastern women with ACS than in men. Increased age, especially post menopause significantly increases CVD risk in women who are normally protected from endogenous estrogens at younger ages. This contributes to differences in risk profiles between men and women already present at admission, differences that explains much of the disparity in outcome. There appears to be gender differences in treatment. Atypical clinical presentation of ACS in women and underestimation of patient risk by the treating physician may be important reasons. Greater awareness of this treatment risk paradox may help to eliminate the gender gap between our current guidelines and management practices. Nevertheless, as most of the differences in outcome are due to baseline characteristics, disparities in outcome may not disappear altogether even if medical management were completely gender blind.
